# Editorial for the Special Issue: Human Pathogenic Filamentous Fungi from Food/Water and Mycotoxins from Water

**DOI:** 10.3390/microorganisms7010021

**Published:** 2019-01-16

**Authors:** R. Russell M. Paterson

**Affiliations:** 1 CEB—Centre of Biological Engineering, University of Minho, 4700-057 Braga, Portugal; russell.paterson@deb.uminho.pt; 2 Department of Plant Protection, Faculty of Agriculture, Universiti Putra Malaysia, Serdang 43400 UPM, Selangor D.E, Malaysia

This special issue was conceived due to the success of the book by Paterson and Lima [1] on human fungal pathogen from food, and a subsequent paper which provided a list of foods from which human pathogenic fungi had been isolated [2]. These were seminal contributions to stimulate interest in to what extent filamentous fungi from food contribute to human mycoses, which are increasing in a very significant manner. The possibility that filamentous fungi from food, and often investigated by food mycologists, may cause human mycoses, studied by medical mycologists, is a novel concept. There is a lack of awareness of each other’s field. The publications [1,2] were conceived to provide a foundation for further study, which could be updated: Paterson and Lima [2] also contains a large table of pathogenic filamentous fungi isolated from foods. The rationale of the special issue was also to provide new data to that provided in [2] which reviewed information only from 2014. 

In this event, the papers we received provided more information than simply contributing to the existing list. Another objective was to produce data on mycotoxins from drinking water but this paper was not forthcoming. However, the current invited editor (RRMP) co-authored a letter, just published, concerning some problematic data on the subject [3] which gives a lead into the literature and may be helpful to interested readers. There remains a need for a review on mycotoxins in drinking water. 

We gratefully accepted a paper on fungal pathogens from water [4]. Although fungi have not widely been applied to water quality regulations, the incidence of fungal infections worldwide is growing, and changes in antimicrobial resistance patterns are taking place. The authors mention that food-related opportunistic filamentous fungi isolated also from water included *Acremonium, Alternaria, Aspergillus, Chaetomium, Fusarium, Mucor, Lichtheimia, Paecilomyces, Penicillium, Phoma, Scopulariopsis,* and *Trichoderma*. A table of ca. 30 taxa are provided that were isolated from water and are human pathogens, not associated with food *per se*. 

It was very gratifying to receive a paper on *Mucor* [5] as one of the more important disease fungi, in an extensive and useful review. They considered the need to develop actionable policies and guidelines regarding dietary restrictions for humans, mycological criteria and specifications for manufacturers, together with potentially enforceable risk-based regulations geared toward safety. Mucormycete transmission through food may occur *via* fungal fermented products relying on *Mucor* and/or *Rhizopus*. 

Moreira et al. [6] draw attention to the ability of *Paecilomyces* to grow at high temperatures, colonize food products, and cause human disease. *Paecilomyces variotii* is the principal agent of food spoilage or contamination of the genus and it is most associated with human hyalohyphomycosis, with clinical manifestations including peritonitis, and cutaneous and disseminated infections, amongst others. However, it is unknown if contaminated foods may be fomites.

*Phoma* spp. have the potential to be pathogenic in humans although it is rare [7]. However, as the immunocompromised population increases, so do the reports of the infections. Species have been isolated from water sources, food and crops; thus acting as potential opportunistic pathogens when a host is exposed. These fungi contaminate common food sources such as potatoes and maize, a common species isolated being *Phoma sorghina*. *Phoma* spp. contaminate seeds, nuts, soybeans, potatoes, bananas, sorghum, maize, kiwi berries, lemons, tomatoes, aubergines, and pomegranates

*Wallemia* of the order Wallemiales (Wallemiomycotina, Basidiomycota), comprise the most xerotolerant, xerophilic and also halophilic species [8]. Species are found in various “osmotically-challenged” environments, such as dry, salted, or highly sugared foods, dry feed, hypersaline waters of solar salterns, salt crystals, indoor and outdoor air, and agriculture aerosols. The fungi are involved in human health problems, as either allergological conditions (e.g., farmer’s lung disease) or rare subcutaneous/cutaneous infections.

In a welcome, predominantly taxonomic paper, which was very detailed, *Acremonium* was described as (a) being regularly isolated from food and (b) a cause of human disease [9]. The authors resolved confusion that has strongly hampered the accurate interpretation of these fungi. The recently-designated type species, *Acremonium alternatum*, is known only from a single isolate, but it is the closest known relative of *Acremonium sclerotigenum/egyptianum*, shown to be most appropriately named as *A. egyptiacum*. This paper is an extensive piece of work that has involved an impressive range of authors and collaborators. 

However, we did not manage to secure a paper on human fungal pathogens isolated from food that have also been reported from drinking water: There remains a requirement for a review on this topic. Nevertheless, an excellent paper on *Aspergillus* in water was published in the special issue [10]. Neither did we manage to obtain papers on *Aspergillus* and *Fusarium* from food that caused human mycoses and there is perhaps a need for an up-to-date review in these areas, although they are covered in the publications [1,2]. Furthermore, there are a large number of genera that are listed in the supplementary file in [2] about which papers were not received, and there is scope for publications on these in the future. 

Finally, special thanks is due to the publishers for their professional assistance at all stages and to the crucially helpful editors, Prof Nelson Lima (Portugal) and Dr Ida Skaar (Norway).

## References

[1] Paterson R.R.M., Lima N. (2015). Molecular Biology of Food and Water Borne Mycotoxigenic and Mycotic Fungi of Humans.

[2] Paterson R.R.M., Lima N. (2017). Filamentous Fungal Human Pathogens from Food Emphasising *Aspergillus*, *Fusarium* and *Mucor*. Microorganisms.

[3] Paterson R.R.M., Buddie A. (2018). Your mycotoxins in water paper. Environ. Sci. Pollut. Res. Int..

[4] Novak Babič M., Zupančič J., Brandão J., Gunde-Cimerman N. (2018). Opportunistic Water-Borne Human Pathogenic Filamentous Fungi Unreported from Food. Microorganisms.

[5] Snyder A., Worobo R. (2018). Risk Mitigation for Immunocompromised Consumers of Mucormycete Spoiled and Fermented Foods: Germane Guidance and Remaining Needs. Microorganisms.

[6] Moreira D., Oliveira M., Borba C. (2018). Human Pathogenic *Paecilomyces* from Food. Microorganisms.

[7] Bennett A., Ponder M., Garcia-Diaz J. (2018). *Phoma* Infections: Classification, Potential Food Sources, and Their Clinical Impact. Microorganisms.

[8] Zajc J., Gunde-Cimerman N. (2018). The Genus *Wallemia*—From Contamination of Food to Health Threat. Microorganisms.

[9] Summerbell R.C., Gueidan C., Guarro J., Eskalen A., Crous P.W., Gupta A.K., Gené J., Cano-Lira J.F., Van Iperen A., Starink M., Scott J.A. (2018). The Protean *Acremonium. A. sclerotigenum/egyptiacum*: Revision, Food Contaminant, and Human Disease. Microorganisms.

[10] Richardson M., Rautemaa-Richardson R. (2019). Exposure to *Aspergillus* in Home and Healthcare Facilities’ Water Environments: Focus on Biofilms. Microorganisms.

